# Detection of *Mycobacterium tuberculosis* Complex Bacilli and Nucleic Acids From Tongue Swabs in Young, Hospitalized Children

**DOI:** 10.3389/fcimb.2021.696379

**Published:** 2021-06-14

**Authors:** Christopher Ealand, Julian Peters, Olivia Jacobs, Astika Sewcharran, Azra Ghoor, Jonathan Golub, Heena Brahmbhatt, Neil Martinson, Ziyaad Dangor, Sanjay G. Lala, Bavesh Kana

**Affiliations:** ^1^ Department of Science and Innovation/National Research Foundation (DSI/NRF) Centre of Excellence for Biomedical TB Research, School of Pathology, Faculty of Health Sciences, University of the Witwatersrand and the National Health Laboratory Service, Johannesburg, South Africa; ^2^ Department of Paediatrics and Child Health, Faculty of Health Sciences, University of the Witwatersrand, Johannesburg, South Africa; ^3^ Center for TB Research, Johns Hopkins University, Baltimore, MD, United States; ^4^ United States Agency for International Development (USAID), South Africa, Pretoria, South Africa; ^5^ Perinatal HIV Research Unit (PHRU), Faculty of Health Sciences, University of the Witwatersrand, Johannesburg, South Africa; ^6^ Paediatric Education and Research Ladder, Department of Paediatrics and Child Health, Faculty of Health Sciences, University of the Witwatersrand, Johannesburg, South Africa

**Keywords:** auramine staining, spoligotyping, qPCR, tongue swabs, pediatric tuberculosis

## Abstract

Diagnosis of tuberculosis in pediatric patients remains challenging due to inherent difficulties associated with obtaining respiratory samples for molecular and culture-based testing. To address this, recent studies have highlighted the utility of tongue swabs to detect *Mycobacterium tuberculosis* genomic DNA in the oral epithelia of tuberculosis infected adults. It is unknown whether tongue swabs have similar utility for diagnosis of childhood tuberculosis and if the presence of DNA in these swabs was associated with whole bacilli. We therefore sought to conduct a preliminary assessment of the utility of tongue swabs to detect tubercle bacilli and their associated genetic material in young children. For this, we recruited hospitalized children with clinically diagnosed tuberculosis (*n* = 26) or lower respiratory tract infection (LRTI, *n* = 9). These categories were blinded for downstream laboratory tests, which included PCR, spoligotyping, smear microscopy, and culture. *Mtb* genomic DNA was detected by PCR only in clinically diagnosed TB cases [11/26 (31.4%)] and not in cases with LRTI. Of these, 5/11 [45.5%] were associated with a spoligotype. Spoligotyping also detected an additional six specimens that were negative by PCR. Using smear microscopy, 19/26 [73.1%] and 4/9 [44.4] were *Mtb* positive in the tuberculosis or LRTI categories respectively. We noted positive results on all three tests in 5/26 [19.2%] in the tuberculosis category and 0/9 in the LRTI category. All specimens were culture negative. Collectively, these preliminary data present a compelling case for broader testing of tongue swabs to diagnose tuberculosis in children where obtaining standard sputum specimens is not easy.

## Introduction

Tuberculosis (TB) continues to cause significant death particularly in high prevalence settings where co-infection with HIV/AIDS has remained one of the primary drivers of infection (WHO, 2019). In addition to people living with HIV, TB infection in other vulnerable populations such as children continues to contribute significantly to the global burden of morbidity and mortality associated with this disease. Pediatric TB has long been neglected from the perspective of appropriate diagnostic tools and child-friendly chemotherapies (Lamb and Starke, 2017; Thomas, 2017; Carvalho et al., 2018; Hatzenbuehler and Starke, 2018; Furin, 2019; Howard-Jones and Marais, 2020). As a result, it is estimated that one million children are infected annually with TB, of which almost half are less than 5 years of age and most are not correctly diagnosed or treated (Jenkins et al., 2014; WHO, 2018; Reuter et al., 2019). A recent WHO report suggested that pediatric TB accounts for 10% of new and relapse TB cases in the African region, relative to the 6.5% incidence observed globally (WHO, 2019). These numbers likely reflect an underestimate as accurate diagnosis of pediatric TB is problematic.

As with adult patients, diagnosis of pediatric TB follows careful assessment of clinical evidence, including a medical examination, TB status of household contacts, clinical history of infection, and results from diagnostic tests [mantoux/tuberculin skin test (TST), chest X-rays, and sputum smear microscopy] (WHO, 2014). Smear microscopy remains the most widely used diagnostic tool, particularly in low- and middle-income settings but offers little utility in young children where it is variable due to the pauci-bacillary nature of infection (Marais and Pai, 2007; Newton et al., 2008; WHO, 2014). Positive smears are typically confirmed by culture or nucleic acid testing. In children, alternative sampling procedures such as gastric aspiration or sputum induction are routinely used despite the fact that they are uncomfortable and potentially produce aerosols, promoting further TB transmission (Zar et al., 2000; Zar et al., 2013). Considering this, easier and safer sampling approaches are required.

Recent studies have demonstrated that *Mycobacterium tuberculosis* (*Mtb*) genomic DNA (gDNA) can be detected *via* oral swabs (either buccal, cheek, or tongue), using a PCR approach, in adults and non-human primates. PCR positivity from oral swabs was detected in 18/20 (90%) confirmed TB cases whereas healthy controls were all negative (Wood et al., 2015). Luabeya et al. showed that tongue swabs yielded superior signal relative to cheek or gum swabs. In a two-phase study, two tongue swabs per patient displayed a combined sensitivity of 92.8% compared to sputum GeneXpert (Luabeya et al., 2019). Similarly, an approach comprising the use of two cheek swabs in children yielded promising findings (Nicol et al., 2019). Here, when compared to confirmed TB, oral swab testing by PCR had a sensitivity and specificity of 43 and 93%, respectively but sensitivity was lower than GeneXpert tests on sputum (64%).

The presence of whole tubercle bacilli on clinical specimens is an important consideration for integrated extraction and diagnostic platforms such as the GeneXpert, which relies on the ability to capture intact TB bacteria during the first washing steps (Blakemore et al., 2010). The presence of free *Mtb* gDNA in swabs, without intact bacteria, may explain the poor performance of swabs on integrated detection platforms suggesting that an alternate approach is required to maximize diagnostic pickup with tongue swabs. To further evaluate this, we set out to determine if PCR-based detection of *M. tuberculosis* gDNA was associated with the presence of whole bacilli and to determine smear positivity in general with tongue swabs. As all these prior data pointed to the superior performance of tongue swabs in adults (Luabeya et al., 2019), we sought to further evaluate this in a small group of hospitalized children to provide preliminary evidence for extending the testing of tongue swab utility for tuberculosis diagnosis in children. We further aimed to assess whether detection of *Mtb* gDNA is associated with the accumulation of intact *Mtb* bacilli in young children and furthermore, that tongue swabs could potentially capture these for downstream testing.

## Materials and Methods

### Study Design and Population

All methods were performed in accordance with approved clinical guidelines. This prospective study (EDICT—Enhanced Diagnostics and Improving Outcomes for Childhood Tuberculosis) was a subsidiary of the ACTTIS study (University of the Witwatersrand HREC; Reference number 172210). Ethics clearance was obtained on the 26^th^ May 2017 (Human Research and Ethic Committee of the University of the Witwatersrand; 170512B). All patients were recruited *via* the Perinatal HIV Research Unit (PHRU) situated at the Chris Hani Baragwanath Academic Hospital (CHBAH) and surrounding areas in greater Johannesburg, South Africa.

### Inclusion Criteria

All parents or legal guardians consented to their children’s participation in the ACTTIS Study before enrollment. Young children (≤5 years of age) had to be clinically diagnosed with TB or lower respiratory tract infection (LTRI)—with the former performed according to the updated and standardized clinical case definitions, including prior TB exposure, chest radiographs, and/or TST findings (Graham et al., 2015). Children with LTRI were those who, although investigated for TB, were not started on TB therapy. The majority of LTRI cases were due to bronchopneumonia or bronchiolitis caused by a variety of bacterial and viral pathogens.

### Exclusion Criteria

Refusal of informed consent and any child who is in an orphanage or institutionalized care as the legal requirements for consent in South Africa for these children are complicated.

### Recruitment of Study Participants

Assent was obtained from children in accordance with local and national regulations. A total of 35 patients were actively recruited between April and November 2019. For every two children with TB, one with LRTI was recruited, but cases were not matched. Enrollments ceased in March 2020 due to the Covid-19 lockdown restrictions in South Africa. Laboratory-based investigators remained blinded to the clinical diagnoses until all test results were certified.

### Sample Acquisition and Processing

A single swab (Copan, 480C) was brushed along the participant’s tongue six to eight times without inducing a cough or gag-reflex. Swabs were collected before or >24 h after gastric aspirate sampling, placed in 3 ml transport media [Middlebrook 7H9 supplemented with OADC (BD) and Tween 80 (MerckSigma)], and stored at 4°C until processing. Upon receiving the sample in a BSL3 facility, it was de-clumped and decontaminated as previously described (Chengalroyen et al., 2016). Separate aliquots were tested by culture, *IS6110*-based nucleic acid amplification, spoligotyping, and smear microscopy.

### Detection of *Mtb* gDNA Using qPCR

Primers designed to amplify the *IS6110* insertion element were used with a minor groove binding (MGB) probe to detect the presence of *Mtb* gDNA (*IS6110*_F2, 5′-GGGTAGCAGACCTCACCTATG-3′, *IS6110*_R2, 5′-AGCGTAGGCGTCGGTGA-3′, *IS6110*_MGB2, 5′-FAM-TCGCCTACGTGGCCTTT-MGBQ-3′ (Savelkoul et al., 2006). PCR cycling conditions were as follows: 45 cycles at 95°C for 30 s, 95°C for 15 s; 60°C for 60 s plus a plate read. A standard curve in the range of 10^6^ to 10^1^ *Mtb* genome equivalents was used to quantify the amount of gDNA in the tongue swab samples. Positive and negative controls for the *Mtb* complex (MTBC) included 100 ng of *Mycobacterium bovis* or *Mycobacterium smegmatis*, respectively. Genomic DNA from tongue swab samples was isolated by incubating 500 µl for 5 min at 95°C following which 8.5 µl of the lysate was used as template for qPCR. We calibrated our PCR testing for *Mtb* genome equivalents using genomic DNA from H37Rv (Massachusetts strain), which contains (16) copies of the IS6110 element (Ioerger et al., 2010). Hence a single genome reflects detection of at least 16 elements. Clinical strains retain up to 25 copies of the IS6110 element (Brosch et al., 2000).

### Genotyping of Putative *Mtb*-Positive Samples With Spoligotyping

DNA extraction was performed by boiling decontaminated swab sample for 5 min. The subsequent lysate was used in a commercially available spoligotyping kit (Ocimum Biosolutions, India) according to methods described in our prior work (Chengalroyen et al., 2016). The resulting data was analyzed using the Spotclust database (http://tbinsight.cs.rpi.edu/run_spotclust.html) according to signatures provided in SpolDB3 and SITVITWEB (Kamerbeek et al., 1997; Brudey et al., 2006; Demay et al., 2012).

### Auramine Smear Microscopy

Ten microliters of each sample was aliquoted onto a standard, glass microscope slide and further spread into a circle of 2 cm in diameter using the pipette tip. This was allowed to air-dry for 30 min inside the biosafety cabinet and then heat-fixed by placing onto a heating block pre-set to 80°C for 30 min. The slides were cooled at room temperature for 5 min and then transferred to a metal rack (over a basin) and flooded with Auramine-O (DMP). Following incubation at room temperature for 20 min, smears were washed using gently running tap-water before de-staining with acid alcohol (DMP) for 2 min. Slides were rinsed as above followed by a counter-stain with Potassium Permanganate (DMP) for 2 min at room temperature. Following a final rinse (as above), smears were air-dried overnight at room temperature. A glass cover-slip (22 × 40 mm) was then placed over each smear and the corners sealed using nail-polish. Slides were removed from the BSL3 facility and placed into the mechanical stage of a fluorescent microscope (Zeiss Observer Z1). Two channels were set up (namely DIC and FITC with exposure times of 100–150 and 3,000 ms, respectively). Spectral properties for FITC excitation and emission were 498 and 526 nm, respectively. The same parameters were used for each sample. A minimum of 100 fields of view were scored for yellow-green, rod-shaped bacteria. A laboratory strain, *Mtb* H37RvS, was used as a positive control.

### Determination of Culturable and Non-Culturable Bacteria Retrieved From Tongue Swab

Our prior data suggested that detection of differentially culturable tubercle bacteria (DCTB) allowed for enhanced recovery of bacteria from sputum using the Most Probable Number (MPN) assay (Chengalroyen et al., 2016). The MPN assay is based on a Poisson distribution of growth in a limiting dilution series. For this assay, 10-fold serial dilutions of decontaminated tongue swab specimens were set up in a final volume of 450 µl, with media that comprised a 1:1 ratio of culture filtrate from an axenic culture of *M. tuberculosis* and fresh Middlebrook 7H9 media. Culture filtrate was added as a source of growth stimulatory molecules to facilitate the recovery of DCTB. For each specimen, the assay was set up in triplicate in 48-well microtiter plates. Un-supplemented, sterile 7H9 media was used as a control. In order to determine the level of culturable bacteria (CFU/ml), decontaminated swab samples were plated on solid media (Middlebrook 7H11, Sigma). All plates were sealed, placed in clear plastic bags, and incubated at 37°C for up to 6 weeks before scoring. The total number of bacteria recovered from the MPN assay was estimated using online software (http://www.wiwiss.fu-berlin.de/fachbereich/vwl/iso/ehemalige/wilrich/index.html).

### Statistical Analysis

The sensitivity and specificity of tongue swab tests using either (i) clinical TB diagnosis or (ii) confirmed TB (*i.e.* culture or GeneXpert positive) as a reference were tested using two-by-two tables and reported as percentages. Associations between positive tongue swab tests and (i) or (ii) were tested using Fisher’s exact test.

## Results

### Demographic Data

**Figure 1:**
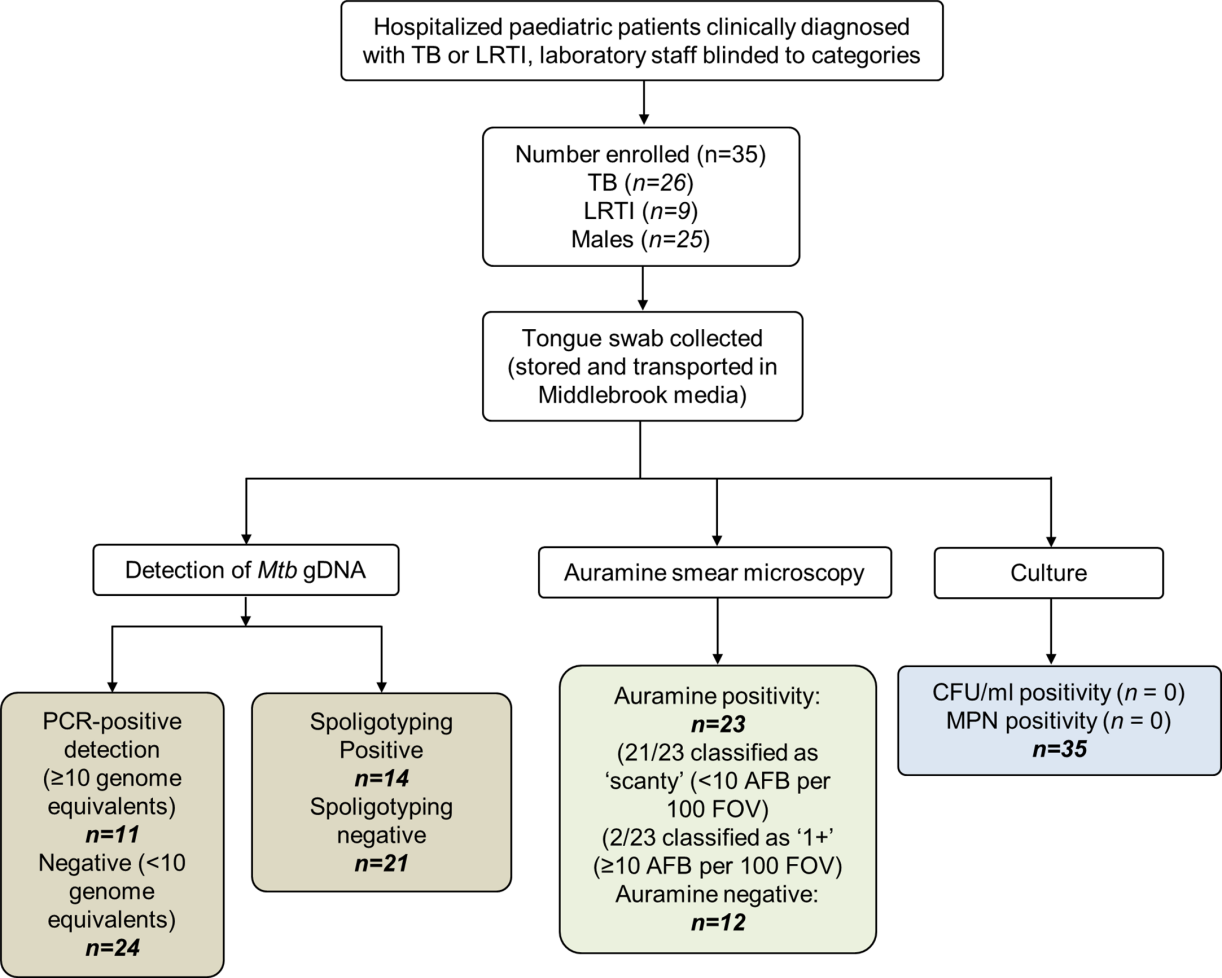
Participant disposition flow chart. A total of 35 patients were analyzed in this study. These children were hospitalized for either tuberculosis (TB) or lower respiratory tract infection (LRTI). The clinical diagnoses were blinded for downstream tests which included detection of genomic DNA (gDNA, using qPCR and spoligotyping), smear microscopy, and culture using detection methods to quantify culturable [colony forming unit (CFUs)] and differentially culturable tubercle bacteria [most probable number (MPN)]. Upon unblinding, 26 patients were grouped into the TB category. Of these, 11 (42.3%) had been detected using PCR genome equivalents (GE) ≥10. Fourteen swabs were positive by spoligotyping. Of the nine patients that were diagnosed with LRTI, a single *Mtb* strain-type and two *Mycobacterium africanum* strains were detected by spoligotyping. TB, tuberculosis; LRTI, lower respiratory tract infection; MPN, most probable number; CFU/ml, colony forming units per ml; AFB, acid-fast bacilli will present as Auramine-positive; FOVs, fields of view; IS6110, insertion element only found within the members of the *Mycobacterium tuberculosis* complex (MTBC); qPCR, quantitative real-time PCR; DR, direct repeats are separated by spacers that polymorphic—unique in the different members of the MTBC.

The participant disposition flow chart is shown in **Figure 1**. Tongue swabs were collected from 35 young children [median age 9.5 months; interquartile range (IQR): 4.4–19.0 months; predominantly male (71.4%)]. Twenty six (74.3%) were clinically diagnosed with TB, of whom six (23.1%) were culture or GeneXpert positive. One child with TB died during hospitalization. Nine children (25.7%) were clinically diagnosed with LRTI. Approximately one quarter (*n = 6*; 23.1%) of children diagnosed with TB was HIV-infected; there were no HIV-infected children in the LRTI group (**Table 1**).

**Table 1:** Demographic characteristics of young children diagnosed with TB or LRTI.

Parameter	Overall *(n = 35)*	Tuberculosis *n = 26 (74.3%)*	LRTI *n = 9 (25.7%)*
Male gender	25 (71.4)	18 (69.2)	7 (77.8)
Age (months)	9.5 (4.4–19.0)	10.2 (5.0–20.0)	9.5 (3.5–11.0)
Weight for age z-score	−1.52 (−2.97 to −0.68)	−1.51 (−2.97 to −1.01)	−2.71 (−2.97 to −0.59)
Height for age z-score	−2.17 (−3.58 to −0.94)	−2.20 (−3.29 to −0.37)	−1.17 (−4.21 to −1.09)
BMI for age z-score	−0.96 (−1.69 to −0.11)	−.98 (−1.69 to −0.31)	−0.27 (−1.39 to +0.12)
HIV infected	6 (17.1)	6 (23.1)	0
HIV exposed uninfected	7 (20.0)	5 (19.2)	2 (22.2)
HIV unexposed	19 (54.3)	13 (50.0)	6 (66.7)
HIV unknown	3 (8.6)	2 (7.7)	1 (11.1)

### Detection of *Mtb* gDNA Using qPCR and Spoligotyping

Based on previous studies wherein *Mtb* gDNA was detected on tongue swabs from adults, we set out to assess if similar utility could be demonstrated using this clinical specimen in hospitalized children. For this, we set up a detection system for the amplification of gDNA from members of the MTBC (*i.e. Mtb* and *M. bovis*) using a previously reported primer and MGB-probe combination (Savelkoul et al., 2006). We found good performance as the PCR test was able to quantitatively detect as little as 10 *Mtb* genomes, with no probe cross-reactivity to gDNA from non-tuberculosis mycobacteria (*i.e. Mycobacterium smegmatis*) (**Supplementary Figures 1A, B** **)**. Based on this, we set our accurate detection limit as ≥10 genome equivalents (GEs) to denote positivity for MTBC gDNA. Using this approach, 11/35 (31.4%) samples were positive for *Mtb* genomic DNA, with the highest in sample 1 (approximately 10^2^ genomes) (**Table 2**). Spoligotyping detected an *Mtb* strain in 12/35 [34.3%] and *Mycobacterium africanum* in 2/35 [5.7%]. Based on clinical diagnosis, 26 individuals were classified with TB infection, PCR, and spoligotyping detected 11/26 [42.3%] positive specimens, but these did not always overlap (**Table 2**). With the PCR positive tongue swabs, 5/11 [45.5%] yielded Fam 33, Haarlem 1, Lam 9, Haarlem 3, and T1 *Mtb* genotypes. In the remaining specimens (with <10 GE), only 7/23 (30.4%) contained *Mtb* strains (2× Beijing, 2× T1, 1× Fam33, 1× Harlem 3 and 1× Vietnamese EAI4/EAI5). Spoligotyping octal code and SpolDB3 genotype are shown in **Supplementary Table 1**. Interestingly, two samples were positive for *Mycobacterium africanum*, a member of the MTBC but were negative for all other tests conducted.

**Table 2:** Detection of *Mycobacterium tuberculosis* complex bacilli and nucleic acids from tongue swabs in young, hospitalized children with clinically diagnosed tuberculosis (“T”; orange) or lower respiratory tract infection (“L”; yellow).

Patient number	Clinical diagnosis	TST Positive	HIV Positive	Culture/GeneXpert	Tongue swab analysis		
					qPCR	Spoligotyping (*M.tb*-specific lineages specified)	Smear Microscopy
1	T				208	Fam 33	
2*	T	ND			51		
3	T	** +**	**+**		46		
4	T	** +**			35		
5	T	** +**			33		
6	T	ND			32		
7	T				25	Haarlem 1	
8	T	ND	**+**		23	Lam 9	
9	T	** +**			14	Haarlem 3	** +**
10	T	** +**	**+**		11	T1/Haarlem 3	
11	T		**+**		10		
12	T				8		
13	T	ND	**+**		6	Beijing	
14	T				5	Beijing	
15	T	ND	ND		5	T1	
16	T				4	T1	
17	T	** +**			4	Haarlem 3	
18	T	** +**			3		
19	T				3		
20	T				3		
21	T	** +**			3		
22	T				3		
23	T				2	EAI4/EAI5	
24	T				2		
25	T	** +**			2		
26	T	** +**			0		
27	L	ND			8		
28	L				8		
29	L				5	Fam 33	
30	L				4		
31	L				3		
32	L		ND		1		** +**
33	L	ND			0	**M. africanum	
34	L				6		
35	L	ND			2	**M. africanum	

Specimens have been categorized in reducing order of Mtb gDNA equivalents as determined by qPCR. Shaded blocks (any color) or “+” indicate positive results. Genome equivalents (per µl) are shown for qPCR results (red); ≥10 genome equivalents were regarded as positive. As per WHO reporting guidelines, two specimens (numbers 9 and 32) had a grading of “1+” positivity for smear microscopy (dark green); all other smear positives were graded as “scanty” (light green). ND, test not done or result unavailable. *Patient #2 died during hospitalization. **Mycobacterium africanum, a MTBC member that is considered a contaminant in our setting, was detected in 2/9 (22.2%) children with LRTI. We were unable to recover Mtb by conventional MGIT or resuscitation factor-enriched culture (Chengalroyen et al., 2016). TST, tuberculin skin test; HIV, human immunodeficiency virus; qPCR, quantitative polymerase chain reaction.

### Smear Microscopy for Acid Fast Bacilli

**Figure 2:**
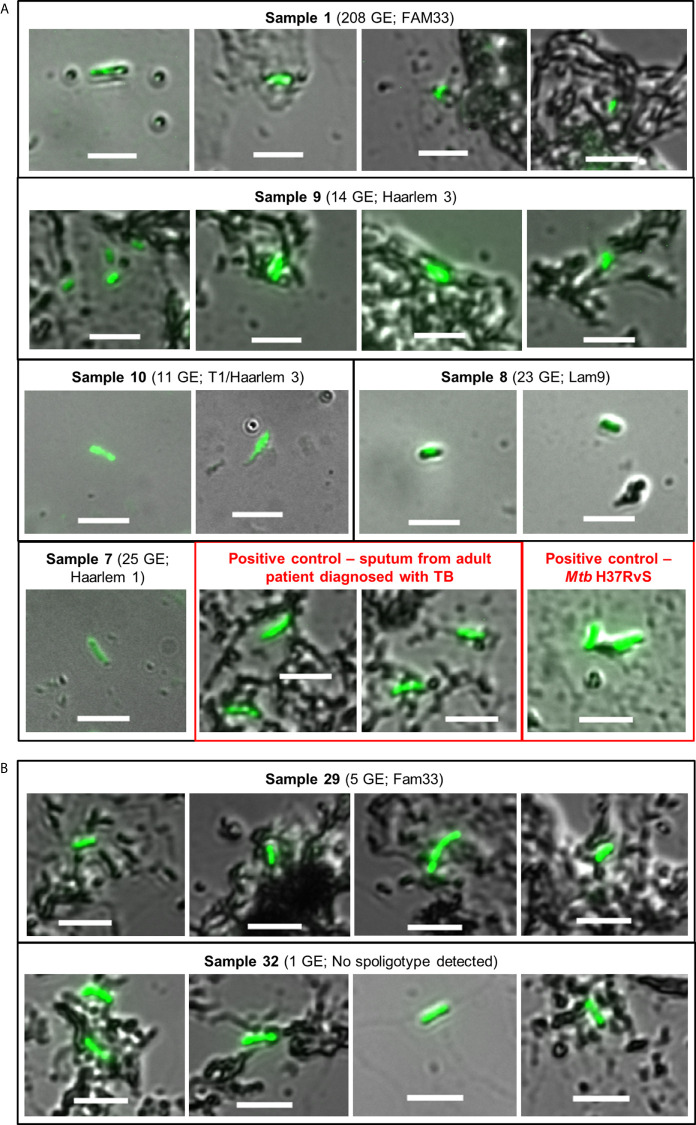
Auramine smear microscopy from tongue swab samples. Ten microliters of each sample was aliquoted onto a microscope slide and stained with Auramine-O. **(A)** Representative images for positive samples from patients clinically diagnosed with TB; containing ≥10 genome equivalents as detected by qPCR and a spoligotype. Boxes in red represent positive controls from sputum (adult patient diagnosed with TB) and the lab strain of *Mtb* (H37Rv). **(B)** Samples from patients clinically diagnosed with LRTI, but their smears were detected as positive for *Mtb*. Sample 29 contained <10 genome equivalents of *Mtb* gDNA, ‘scanty’ acid-fast bacilli count and was assigned a strain-type by spoligotyping. Sample 32 was scored as ‘1+’ for acid-fast bacilli but negative for all other tests. In all cases, slides were viewed using a Zeiss Observer Z1 using two channels, *i.e.* DIC (bright-field) and FITC (green) with exposure times of 100–150 and 3,000 ms, respectively. Scale bar represents 5 µm.

Having established that tongue swabs were practical for detection of gDNA, we next sought to determine how this compared to smear microscopy and if the detection of gDNA correlated with the presence of whole *Mtb* bacilli using Auramine smear microscopy. Acid-fast bacilli (AFB) were detected in 23/35 [65.7%] tongue swabs collected; of these positive *Mtb* smears were detected in 19/26 [73.1%] tongue swabs in the group clinically diagnosed with TB and 4/9 [44.4%] in individuals clinically classified as LRTI (**Table 2**). Despite high positivity on smear, very few AFB (<10) were detected, with most samples classified as ‘scanty’. Only 1/11 (9.1%) specimen (patient 9) scored as ‘1+’ (**Figure 2A** and **Table 2**). In the group clinically diagnosed with LTRI, 4/9 (44.4%) were classified as ‘scanty’ for AFB and one (11.1%) (patient 32) was classified as ‘1+’ (**Figure 2B** and **Table 2**).

### Assessment of Culturable and Non-Culturable Bacteria

Following the observation that whole AFB could be detected from tongue swabs, we next sought to establish whether samples contained viable *Mtb* as culture remains the gold standard for laboratory test for the diagnosis and management of TB. A positive culture may result from as little as 10 bacilli per milliliter of specimen (Highsmith et al., 2019). We utilized the MPN assay for resuscitation of DCTB (Chengalroyen et al., 2016) and plating on solid media to determine culturable CFU/ml. In both cases, the level of viable bacteria retrieved from the tongue swab could not be determined as no growth was detected in any of the assays used in this study (data not shown). It should be noted that the primary diagnostic result, based on gastric lavage, yielded culture or GeneXpert positivity on 6/26 [23.1%] individuals classified as infected with TB.

### Comparative Analysis of *Mtb* Detection Using Different Approaches

Given that tongue swabs yielded varying degrees of positive detection using different laboratory tests, we next sought to compare *Mtb* detection across different assays (**Table 2**). Of tongue swabs positive by PCR, 10/11 were also smear positive [90.9%] and 5/11 [45.5%] were positive with spoligotyping. No PCR positivity was detected in cases with LRTI, suggesting superior specificity of this approach. In PCR negative tongue swabs, 13/24 [54.2%] yielded a positive smear while 9/24 [37.5%] yielded a spoligotype (albeit with two *M. africanum* cases). When overlaid with the clinical diagnosis (26 TB and nine LRTI cases), 5/26 [19.2%] specimens were positive on all three tests whilst 9/26 [34.6%] were positive on spoligotyping and smear. Interestingly, 21/26 [80.8%] specimens were positive on one or more tests. With clinically classified LRTI, 1/9 [11.1%] specimens was positive on one or more tests.

## Discussion

Children continue to be incorrectly diagnosed with TB leading to inappropriate management which widens the case-detection gap and fuels transmission, particularly in high-burden settings with poor health resources (Furin, 2019; Howard-Jones and Marais, 2020). Despite being less contagious than adults, the risk of progressing to active TB is greater in infants and children compared to adolescents (Holmberg et al., 2019). Pediatric TB is more difficult to diagnose because primary infection is not characterized by clear symptoms, and IGRAs or TSTs are less reliable (Loeffler, 2003). Disease often manifests as pauci-bacillary and can be either pulmonary or extra-pulmonary disease (Piccini et al., 2014; Highsmith et al., 2019). In addition, children tend to swallow respiratory secretions necessitating other uncomfortable procedures such as gastric lavage or sputum induction (Velayati, 2016; Kay et al., 2020). There is therefore an urgent need to improve TB diagnosis in children and/or to develop improved diagnostic tools, together with actively exploring the testing of new, non-invasive specimen types. Recent studies have shown that *Mtb* gDNA can be retrieved from the oral cavity of adults with high PCR specificity (Wood et al., 2015; Luabeya et al., 2019) and from cheek swabs in children (Nicol et al., 2019). The promising performance of tongue swabs in adults suggested that further studies in children using this specimen could be useful.

Herein, we utilized tongue swabs in combination with quantitative PCR specific for members of the MTBC, spoligotyping, culture, and smear microscopy. Thirty five tongue swabs were obtained from young (≤5 years of age), hospitalized children, and this preliminary study shows that this alternative sample type was positive for *Mtb* more frequently in children with clinically diagnosed TB than non-TB LRTI. This is consistent with studies reporting detection of *Mtb* from oral or tongue swab scrapings from adults with TB (Wood et al., 2015; Luabeya et al., 2019; Mesman et al., 2019; Nicol et al., 2019). From our data, smear microscopy appears to yield the greatest diagnostic pickup. However, given the inherently poor performance of smear and the fact that most smears were scanty, combination of this technique with molecular tests such as PCR may yield maximum benefit from tongue swabs, an approach that should be validated in larger studies. In support of this, we had two samples that were negative by smear but *Mtb* positive using spoligotyping.

In tongue swab specimens that harbored ≥10 genome equivalents (10/26) (and clinically diagnosed with TB), all were positive in smear microscopy but were scored as ‘scanty’ with <10 acid-fast bacilli detected in at least 100 fields of view. Only one specimen was scored as ‘1+’ and this is reflective of the pauci-bacillary nature of pediatric TB (Piccini et al., 2014; Kendall, 2017) and the low sensitivity of smear testing. In 11/26 (42.3%) of patients diagnosed with TB, a positive spoligotype and an *Mtb* strain-type were detected. In one patient that was clinically diagnosed with LRTI, our tests detected a positive, ‘scanty’ smear and a strain-type (Fam33) by spoligotyping—likely reflective of a clinical misdiagnosis.

The GeneXpert integrated *Mtb* molecular diagnostic platform by first capturing whole bacteria, followed by washing steps to remove all extracellular material. Thereafter, the captured bacteria are lysed to release DNA. Considering this, the presence of whole organisms in tongue swab specimens should be considered when evaluating these specimens in larger studies with platforms such as the GeneXpert and should consider comparisons with tests that also allow for the detection of free *Mtb* gDNA from clinical specimens. Our data suggest that whole organisms can be found in majority of tongue swabs from individuals clinically classified as being infected with *Mtb*. However, no viable bacteria could be detected for auramine-positive samples grown either on solid or liquid media supplemented with growth factors. The reason for this remains unclear and may require further study. Future work should consider multiple scrapings of the tongue to increase bacterial (or DNA) yield.

A positive aspect of this is that the demonstration of the ability to detect TB irrespective of bacterial viability reduces biosafety concerns. Collectively, our preliminary analysis has clearly shown that together with gDNA, intact *Mtb* bacilli can be detected from tongue swabs in young children. A diagnostic approach combining currently available diagnostic tools with tongue swab testing holds great potential for pediatric TB and should be explored further.

## Data Availability Statement

The original contributions presented in the study are included in the article/**Supplementary Material**. Further inquiries can be directed to the corresponding authors.

## Ethics Statement

The studies involving human participants were reviewed and approved by the Human Research and Ethic Committee of the University of the Witwatersrand. Written informed consent to participate in this study was provided by the participants’ legal guardian/next of kin.

## Author Contributions

CE performed experiments, curated and analyzed the data, wrote the original draft, reviewed and edited the final draft. JP performed experiments, curated and analyzed data. OJ performed experiments. AS preformed experiments. AG collected samples at clinical site and made clinical diagnoses. JG provided critical review of manuscript. HB provided critical review of the manuscript. NM conceptualized and designed the study, provided funding to obtain samples and critical review of manuscript. ZD provided critical review of manuscript. SL conceptualized and designed the study, organized samples, curated and analyzed data, drafted, reviewed and edited final manuscript. BK conceptualized and designed the study, supervised the project, provided funding, drafted, reviewed and edited the final manuscript. All authors contributed to the article and approved the submitted version.

## Funding

US Civilian Research and Development Foundation (CRDF Global), Agreement # DAA3-17-63192-1. This publication is based on work supported by a grant from the US Department of Agriculture. BK and CE were supported by the Department of Science and Innovation, the National Research Foundation and the South African Medical Research Council (with funds from the Department of Health). Any opinions, findings and conclusions or recommendations expressed in this material are those of the authors and do not necessarily reflect the views of the US Department of Agriculture.

## Conflict of Interest

The authors declare that the research was conducted in the absence of any commercial or financial relationships that could be construed as a potential conflict of interest.
