# Supplementary material for: Detection of Mycobacterium tuberculosis Complex Bacilli and Nucleic Acids From Tongue Swabs in Young, Hospitalized Children
Source: Front Cell Infect Microbiol. 2021 Jun 14;11:696379. doi: 10.3389/fcimb.2021.696379 (PMC8238041; doi:10.3389/fcimb.2021.696379)
Supplement: Supplementary file 1 [file DataSheet_1.pdf]

Detection of *Mycobacterium tuberculosis* complex bacilli and nucleic acids from tongue swabs in young, hospitalized children

Christopher Ealand[1], Julian Peters[1], Olivia Jacobs[1], Astika Sewcharran[1], Azra Ghoor[2], Jonathan Golub[3], Heena Brahmbhatt[4, 5], Neil Martinson[3, 5], Ziyaad Dangor[6], Sanjay G Lala[5, f6 Φ] & Bavesh Kana[1, †]

[1]DSI/NRF Centre of Excellence for Biomedical TB Research, School of Pathology, Faculty of Health Sciences, University of the Witwatersrand and the National Health Laboratory Service, Johannesburg, 2000, South Africa

[2]Department of Paediatrics and Child Health, Faculty of Health Sciences, University of the Witwatersrand, Johannesburg, South Africa.

[3]Center for TB Research, Johns Hopkins University, Baltimore, Maryland

[4]USAID, South Africa, 100 Totius Street, Groenkloof, Pretoria 0027

[5]Perinatal HIV Research Unit (PHRU), Faculty of Health Sciences, University of the Witwatersrand, Johannesburg, South Africa.

[6]Paediatric Education and Research Ladder, Department of Paediatrics and Child Health, Faculty of Health Sciences, University of the Witwatersrand, Johannesburg, South Africa.

†Correspondence: *Mailing address:* DST/NRF Centre of Excellence for Biomedical TB Research, National Health Laboratory Service, P. O. Box 1038, Johannesburg 2000, South Africa. Phone: Tel: + (27) 11 4899135; Fax: + (27) 11 4899397; *E-mail:* Bavesh.Kana@wits.ac.za

[Φ]Co-corresponding author: *Mailing address*: Paediatric Education and Research Ladder, Department of Paediatrics, Chris Hani Baragwanath Academic Hospital, P O Bertsham, 2013, South Africa *Phone*: Tel: +27 (0)11 933 9781; Fax: +27 (0)865 534 599; *Email*: Sanjay.Lala@wits.ac.za.

Running title: Detection of tuberculosis in children

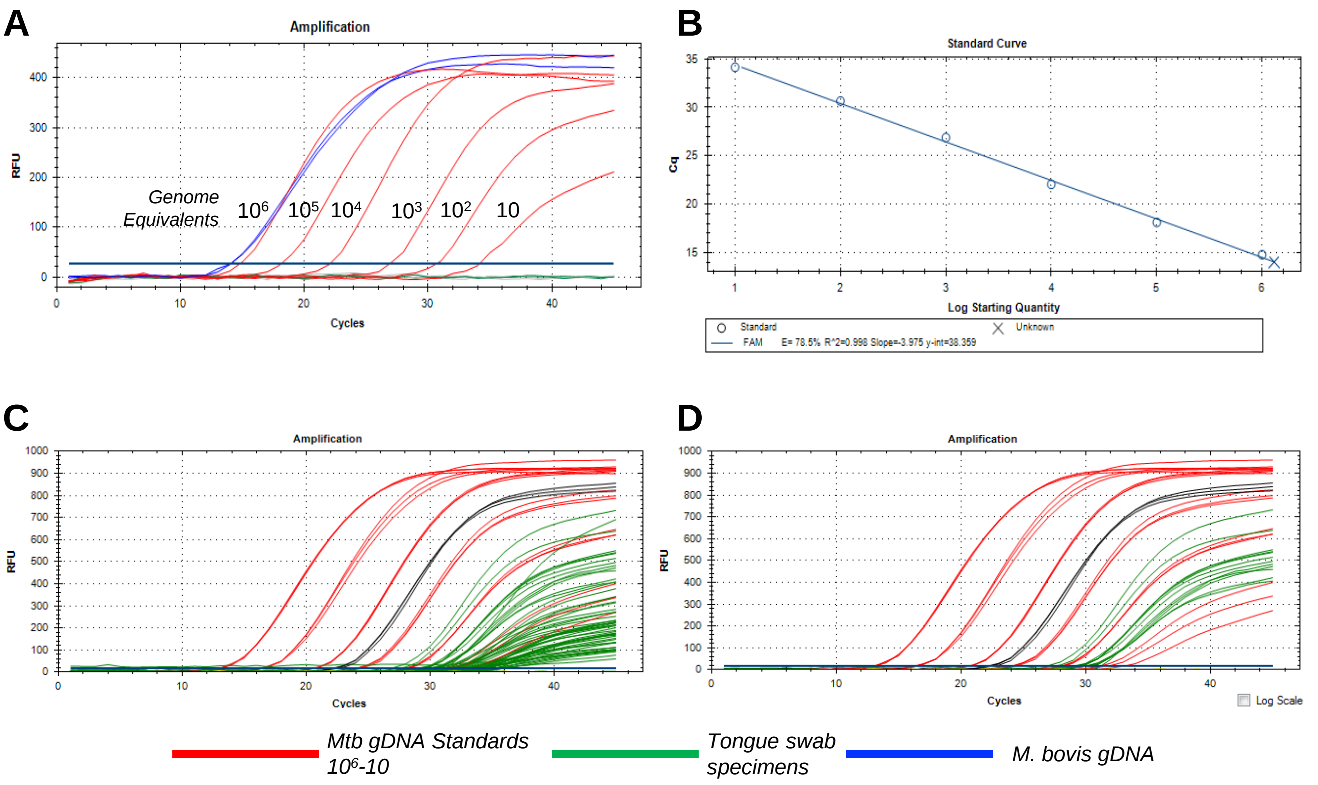

Supplementary Figure 1. Optimization of qPCR primers and MGB-probe to specifically amplify *IS6110* insertion element in gDNA from members of the *Mycobacterium tuberculosis* complex (MTBC). (A) Red lines correspond to amplification plots using *Mtb* H37Rv genomic DNA as a standard curve (10^6 – 10 genome equivalents), blue line corresponds to 100ng of *M. bovis* genomic DNA (positive control), green line corresponds to *Mycobacterium smegmatis* mc^{2}155 (negative control) and grey line corresponds to no template control – no amplification observed in either control sample. **(B)** Standard curve generated off decreasing amounts of gDNA. **(C)** Amplification of *IS6110* element in tongue swab samples (green lines), samples 1 – 31. **(D)** As for per (C) but with remaining samples 32-35. All standards were performed in triplicate while tongue swab samples were performed in duplicate. Genomic DNA was calculated as genome equivalents per µl.

Supplementary Table 1. Strain types identified from in paediatric tongue swabs using spoligotyping

Specimen number	Spoligotyping octal code	SpolDB3 genotype
1	771357777763771	Fam 33
7	511047014000261	Haarlem 1
8	771047606100361	LAM 9
9	511047674500261	Haarlem 3
10	711045674700261	T1/Haarlem 3
13	000000000003771	Beijing
14	000000000003771	Beijing
15	777757675760771	T1
16	777757675760771	T1
17	511047014000261	Haarlem 3
23	713246076303661	EAI4/EAI5
29	771357777763771	Fam 33
33	700007616771661	*Mycobacterium africanum*
35	700007616771661	*Mycobacterium africanum*

Five hundred microliters of each decontaminated tongue swab sample was boiled to extract genomic DNA. The resulting lysate was strain-typed using commercially available kit – amplified DNA was then hybridized to a set of 43 oligonucleotide probes by reverse line blotting on a membrane. The presence of spacers was visualized on an x-ray film as black squares after incubation with streptavidin-peroxidase and detected with enhanced chemoluminescence (ECL) detection solutions. The results were recorded as the presence or absence of spacers using a binary code (B). Spoligotypes were then analyzed using TBlineage Spotclust to assign mycobacterial lineages and/or families according to signatures provided in SpolDB3 and SITVITWEB databases (http://tbinsight.cs.rpi.edu/run_spotclust.html).
